# HPV Infection, but Not EBV or HHV-8 Infection, Is Associated with Salivary Gland Tumours

**DOI:** 10.1155/2015/829349

**Published:** 2015-11-05

**Authors:** Maja Hühns, Georg Simm, Andreas Erbersdobler, Annette Zimpfer

**Affiliations:** ^1^Institute of Pathology, University of Rostock, Strempelstrasse 14, 18055 Rostock, Germany; ^2^Institute of Pathology, University Medical Centre Jena, Ziegelmühlenweg 1, 07743 Jena, Germany

## Abstract

Benign and malignant salivary gland tumours are clinically heterogeneous and show different histology. Little is known about the role of human herpes virus 8 (HHV-8), Epstein-Barr virus (EBV), and human papillomavirus (HPV) infection in salivary gland neoplasms. We investigated the presence of the three viruses in formalin-fixed, paraffin-embedded tissue samples in a cohort of 200 different salivary gland tumours. We performed EBV-LMP-1 and HHV-8 and p16 immunohistochemistry, a specific chip based hybridization assay for detection and typing of HPV and a chromogenic in situ hybridization for EBV analysis. Only one case, a polymorphic low-grade carcinoma, showed HHV-8 expression and one lymphoepithelial carcinoma was infected by EBV. In 17 cases (9%) moderate or strong nuclear and cytoplasmic p16 expression was detected. The HPV type was investigated in all of these cases and additionally in 8 Warthin's tumours. In 19 cases HPV type 16 was detected, mostly in Warthin's tumour, adenoid cystic carcinoma, and adenocarcinoma NOS. We concluded that HHV-8 infection and EBV infection are not associated with salivary gland cancer, but HPV infection may play a role in these tumour entities.

## 1. Introduction

Benign and malignant salivary gland tumours belong to rare head and neck tumours. Most cases are benign and only 20% are malignant [1]. The majority of the diseases arise in the sixth decade and the sex distribution is equal [2]. Salivary gland tumours show a wide range of phenotypic, biological, and clinical heterogeneity [3]. They occur in the major and minor salivary glands, whereof 80% of major salivary gland tumours are present in the parotid glands, and less than half of these tumours are malignant [3].

In general, several viruses have been demonstrated to be the trigger of neoplastic diseases of the head and neck, like human papillomavirus (HPV) [4] and different human herpes viruses, like human herpes virus-4/Epstein-Barr virus (EBV) [5], cytomegalovirus (CMV) [6], and human herpes virus 8 (HHV-8) [7]. However, the role of viruses in the genesis of salivary gland tumours remains still debatable.

The aim of the present study was to determine the prevalence of different pathogens like HHV-8, EBV, and HPV in a large cohort of salivary gland tumours. The involvement of these three pathogens was analyzed by immunohistochemistry, a specific chip based hybridization assay, or chromogenic in situ hybridization.

## 2. Material and Methods

### 2.1. Patients

A total of 200 patients with salivary gland tumours were included, diagnosed between 1990 and 2014 (Table 1). Among those there were 93 malignant and 107 benign tumours of different entities (Table 2). The formalin-fixed, paraffin embedded specimens were retrieved from the archive of the Institute of Pathology at the University Medicine of Rostock.

The study was performed with internal review board approval and patients' data were anonymized in accordance with German laws concerning safety data.

### 2.2. Clinical Data

Clinical data were obtained by reviewing the charts of the Clinical Cancer Registry, University of Rostock. These data were anonymized and included sex, age at diagnosis, and stage (Tables 1 and 2).

### 2.3. Construction of Tissue Microarrays (TMA)

For TMA construction, a hematoxylin and eosin (H&E) stained slide from each block was used to define the representative tumour and normal region by an experienced pathologist. Tissue cylinders with a diameter of 1.0 mm were punched from the specimen block and brought into an empty paraffin block [8] by using a precision instrument (Beecher Instruments, Silver Spring, MD, USA). Three tissue cylinders of tumour and one cylinder with normal tissue from every specimen were prepared. Taken together, 10 different TMA blocks with malignant and benign specimens were constructed.

### 2.4. Immunohistochemistry

For each TMA block, four-micrometer sections were transferred to an adhesive-coated glass slide system (Instrumedics Inc, Hackensack, NJ, USA) and stained with H&E. Only cases containing at least 10% tumour tissue were further analyzed.

Immunohistochemical staining was performed with an autostainer (EnVision FLEX, High pH, (Link), DAKO, Hamburg, Germany) according to the manufacturer's standard protocol with primary antibodies against Cytokeratin AE1/AE3 (monoclonal mouse, reference number: C1702C01, titre 1 : 500, DCS, Hamburg, Germany), EBV-latent membrane protein-1 (LMP-1) (monoclonal mouse, clones CS.1–4, “ready to use,” Dako, Hamburg, Germany), HHV-8 (monoclonal mouse, 1 : 50, reference number 6011336, Leica, Wetzlar, Germany), and p16 (monoclonal mouse, clone G175-405, 1 : 20, BD Biosciences, Heidelberg, Germany).

Cytokeratin AE1/AE3 immunohistochemistry served as a positive control for the tissue studied and proved the stainability of the archival material.

For LMP1 of EBV the presence of unequivocal membranous and cytoplasmatic staining in >2% of tumour cells was considered positive. For HHV-8 the presence of nuclear immunoreactivity in >2% of tumour cells was considered positive. Positivity for p16 was considered when a moderate to strong staining was observed in the cytoplasm and in the nucleus. The staining intensities were graded as negative, weak, moderate, and strong by visual inspection by an experienced pathologist. Weak and patchy p16 signalling was judged as negative. For positive controls, (tumour) tissues with known marker expression were carried along. The positive controls were as follows: HHV8: Kaposi sarcoma in an AIDS patient; EBV-LMP-1: tonsil with a known infectious mononucleosis (EBV positive); EBER-CISH: tonsil with a known infectious mononucleosis (EBV positive); P16: carcinoma of the tonsil with a known HPV infection.

In the negative control experiments, the primary antibodies were omitted.

### 2.5. Detection of EBV

Chromogenic in situ hybridization (CISH) staining for EBV encoded RNA (EBER) transcripts was performed on 4 *μ*m deparaffinized tissue sections, mounted on adhesive coated glass slides according to manufacturer's instructions (ZytoVision, Bremerhaven, Germany). Slides were digested with pepsin solution for 10 minutes, incubated with biotin-labelled ZytoFast EBV probe for 60 minutes at 55°C, followed by incubation with AP-streptavidin for 30 minutes at 37°C, and colouring was performed with NBT/BZIP at 37°C for maximal 40 minutes. Cells exhibiting nuclear staining were considered positive. Positive and negative controls were included in each run.

### 2.6. Identification of HPV Types

The cases selected for molecular HPV analysis were all tumours with moderate or strong cytoplasmic and nuclear p16 expression and additionally in 8 Warthin's tumours. Tumour DNA was extracted from formalin-fixed, paraffin embedded sections in 25 salivary gland cancers with the ReliaPrep FFPE gDNA Miniprep system (Promega, Mannheim, Germany) according to manufacturers' instruction. For identification of HPV types the HPV Type 3.5 LCD-Array Kit (Chipron, Berlin, Germany) was used according to manufacturer's instructions. Briefly, two PCR reactions were performed using supplied My11/09 (product size 450 bp) and “125” (product size 125 bp) primer mixes and analyzed by agarose gel electrophoresis. Both PCR products were mixed and hybridized on the 3.5 LCD chip slide. The slide was subsequently scanned on the Slide Reader Scanner and evaluated with the Slide Reader Software (Chipron, Berlin, Germany).

## 3. Results

### 3.1. Patient and Tumour Characteristics

We analyzed 200 patients with malignant or benign salivary gland tumours, diagnosed between 1990 and 2014 (Table 1). The mean age at diagnosis was 58.9 years (range 11–95 years), 107 were male (53.5%), and 93 were female (46.5%). Different tumour entities were included, whereas in malignant tumours mainly mucoepidermoid carcinoma (18.3%), adenoid cystic carcinoma (17.2%), and adenocarcinoma NOS (10.8%) occurred. In benign tumours basically Warthin's tumours (43%) and pleomorphic adenoma (33%) were diagnosed.

### 3.2. Morphological Evaluation of the Specimens

190/200 (95%) of the specimens contained >10% tumour tissue. Ten cases (5%) had limited tumour tissue and were excluded from the study.

### 3.3. Expression and Detection of HHV-8, EBV, and HPV by Immunohistochemistry

**Figure 1:**
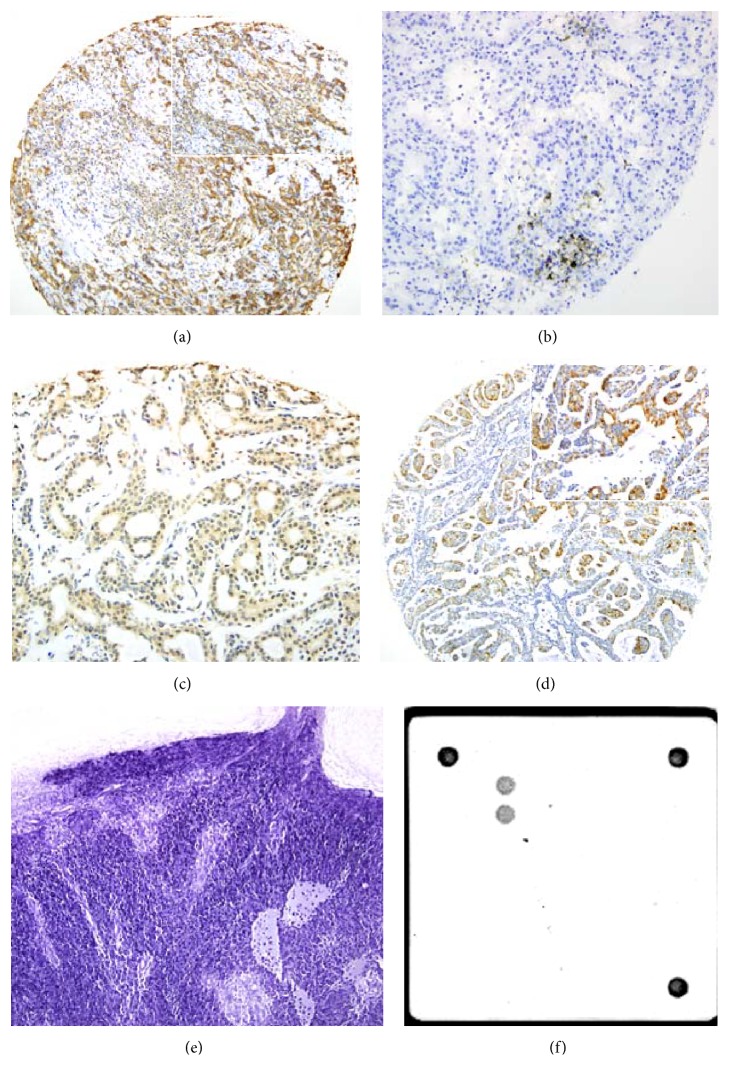
Evidence of pathogens in different salivary gland tumours. (a–d) Immunohistochemistry of (a) AE1/AE3 (10x magnification, in insert 20x magnification) in an adenocarcinoma NOS; (b) HHV-8 (20x magnification) in a polymorphic low-grade carcinoma; (c) EBV-LMP-1 (20x magnification) in a lymphoepithelial carcinoma; and (d) p16 expression (10x magnification, in insert 20x magnification) in an adenoid cystic carcinoma. (e) Lymphoepithelial carcinoma showing EBER expression (10x magnification). (f) Adenoid cystic carcinoma with HPV type 16 detected by Chipron LCD array.

AE1/AE3 positivity was found in 183/190 (96.3%) cases, indicating that most tumours were of epithelial origin and were suitable for further investigations (Figure 1(a)).

Only one case, a polymorphic low-grade carcinoma exhibited HHV-8 expression, located in the nucleus (Figure 1(b)).

A small subfraction of only 4 cases expressed the EBV-LPM-1 protein and just one case, a lymphoepithelial carcinoma, was positive in EBER-CISH analyses (Figures 1(c) and 1(e)).

Moderate or strong nuclear and cytoplasmic p16 was seen in 17/190 (9%) cases (Figure 1(d)). The most frequent tumour type with positive nuclear expression was adenoid cystic carcinoma, followed by adenocarcinoma and acinus cell carcinoma.

### 3.4. Detection of HPV

Detection of HPV types was performed in all 17 p16 nuclear positive cases and also in 8 Warthin's tumours. In 19/25 cases (76%) HPV type was classified (Table 3); 6 cases were negative or DNA was not amplifiable. In all 19 positive cases HPV type 16 was detected (Figure 1(f)) in different tumour entities (Table 4), mostly in malignant cancer types (12/25) compared to benign tumours (7/25).

## 4. Discussion

The participation of viruses in salivary gland tumours is receiving increasing interest. The role of EBV in lymphoepithelial carcinomas (LEC), nasopharyngeal carcinomas, and also benign Warthin's tumours was reported in several studies [9, 10]. In our study, we investigated different salivary gland tumours, including 46 Warthin's tumours and two cases of LEC. But EBV was detected only in one LEC (Figures 1(c) and 1(e)). LEC is a rare malignancy and only few cases with EBV infection were described in the literature. It occurs mainly in East Asia population and only rarely in western countries (reviewed in [11]). However, there are many controversies about the association of EBV infection with LEC. Some studies described negative results [12–17], while other authors found a positive association [11, 18–20]. On the basis of our data of only two cases, we are not able to conclude whether EBV is associated with LEC or not. The presence of EBV in Warthin's tumour was described in several studies, with a ratio of about 20% [9, 21, 22]. However in our study no tumour of this entity showed any positivity for EBV. The present results indicate that infection with EBV does not play a major role in salivary gland neoplasm.

HHV-8, which naturally infects only humans, is known to be involved in various malignancies including Kaposi's sarcoma, Castleman's disease, and primary effusion lymphoma [23–26]. Nevertheless, the role in salivary gland tumours is still unclear. Klussmann et al. reported that HHV-8 has no major tropism to salivary gland epithelium in immunocompetent patients [27]. However, they detected HHV-8 in a bilateral MALT-lymphoma of the parotid gland of a HHV-8 seropositive female patient suffering from Sjögren's syndrome [27]. Other reports found out that HHV-8 is uncommon in the saliva of healthy persons but was found in patients with Kaposi's sarcoma [28, 29]. Klussmann et al. concluded that a latent infection of the salivary gland in people from areas with low prevalence of Kaposi's sarcoma is rare [27]. In contrast, in a Greek study HHV-8 was detected in 44% of Warthin's tumour [7]. Our findings differ from the Greek study, since we detected no HHV-8 in our 46 investigated Warthin's tumours. Only in one adenocarcinoma NOS the virus was found.

p16 is a cyclin-dependent kinase inhibitor of CDK4 and CDK6, which activates the negative cell cycle regulator retinoblastoma protein (pRB). This protein in turn downregulates p16 expression. The human papillomavirus oncogene E7 interferes with pRB and inactivates the protein, resulting in overexpression of p16 [30]. Our results showed in 17/190 cases (9%) a nuclear expression of the surrogate marker p16. Nevertheless, p16 is not a specific marker for the detection of HPV [31] and its value in the detection of HPV infection is still controversial [32]. Due to the role of p16 in cell cycle, we selected only the cases with strong nuclear p16 expression for further HPV typing by DNA analysis. Based on all investigated cases, 19 cases were HPV positive (10%). In several studies, a correlation of HPV infection, mainly types 16 and 18, with salivary gland tumours was shown. In American patients with mucoepidermoid carcinoma 36% were positive, predominantly type 16 and less commonly HPV type 18 or types 16 and 18 together [33]. In two other studies similar results were shown for salivary gland tumours. Hafed et al. detected positivity for HPV type 16 and/or type 18 in 23.5% [34], whereas Lin et al. found 35.8% positive HPV cases [9]. In contrast, in other studies with Warthin's tumours no HPV-positive case could be found [32, 35]. In our cohort of salivary gland carcinomas we identified 6 Warthin's tumours infected with HPV type 16, after preselection by p16 expression, according to 13.3% of all investigated Warthin's tumours.

Most positive HPV cases in our study derived from malignant salivary tumours like adenoid cystic carcinoma and adenocarcinoma NOS and, to a lower proportion from acinus cell carcinoma, salivary duct carcinoma and adenoid basal-cell carcinoma. Recent studies also showed conflicting results with malignant tumours. In some studies with up to or more than 100 salivary gland neoplasms, no HPV positive cases were identified [32, 35–37]. In contrast, other studies showed a presence of HPV in these tumour entities [9, 33, 34].

Besides smoking and alcohol consumption, HPV represents another risk factor for squamous head and neck tumours [38]. High-risk (hr) HPV types, often type 16, were frequently detected in oropharynx carcinoma, mainly in tonsillar carcinoma and tongue base carcinoma [38, 39]. Recent advances in HPV-induced cervical precancerous lesions highlight that different pathways of HPV infection (i.e., viral persistent, transient or latent infection, productive or permissive infection) may or may not progress to a “transforming infection,” with risk of developing high grade squamous intraepithelial lesion and consecutive invasive cancer [40]. The data show that only a minority of hr HPV infections become “transforming infections,” characterised by altered gene expression, especially 2 viral genes E6 and E7 (as discussed above) [40]. Among hr HPV types, type 16 is obviously associated with the greatest risk to develop cervical cancer if left untreated [40, 41]. But which factors determine malignant progression is still poorly understood [40]. And for hr HPV positive head and neck tumours, especially oropharyngeal cancers, data concerning the development and progression of “transforming” hr HPV infections are still limited [26].

Risk factors, like several sexual partners and oral practices, were similar to those of HPV-associated cervix carcinoma [42, 43]. In a recent study, the age for oral HPV infection was dated between 14 and 69 years [44] and is consistent with our results of HPV positive cases (between 31 and 72 years). Due to the growing rates of salivary gland tumours and positivity of HPV types 16 and 18, some studies hypothesized that use of the two available HPV vaccines for cervix carcinoma may cause a reduction of salivary gland cancer [42, 44–46].

## 5. Conclusion

Salivary gland neoplasm is an uncommon and heterogeneous disease. These tumours are not generally associated with EBV or HHV-8 infection. In our investigations we found a correlation between p16 nuclear overexpression and high-risk HPV infection in salivary gland neoplasm. HPV type 16 was identified in adenoid cystic carcinoma, adenocarcinoma NOS, and Warthin's tumours and, to a lesser extent, in acinus cell carcinoma, salivary duct carcinoma, and adenoid basal-cell carcinoma. HPV seems to be involved in a significant proportion of salivary gland tumours but its exact role is still controversial due to the recent studies.

## Figures and Tables

**Table 1:** Patient characteristics of salivary gland tumours.

Clinical characteristics	*n* = 200
Median age, years	59.9
Range	11–95
Sex	
Male	107 (53.5%)
Female	93 (46.5%)
Tumour location	
Parotid gland	175 (87.5%)
Left side	84 (48%)
Right side	69 (39.5%)
Submandibular gland	14 (7%)
Left side	7 (3.5%)
Right side	7 (3.5%)
Minor salivary glands	11 (5.5%)
Stage at presentation (malignant)	
I	36 (38.7%)
II	18 (19.4%)
III	20 (21.5%)
IV	17 (18.3%)
No stadium determinable	2 (2.2%)

**Table 2:** Tumour characteristics of malignant and benign tumours (*n* = 200).

	Type of tumour	Number of cases	Frequency in %
Malignant	Mucoepidermoid carcinoma	17	18.3
	Adenoid cystic carcinoma	16	17.2
	Adenocarcinoma NOS	10	10.8
	Salivary duct carcinoma	9	9.7
	Acinus cell carcinoma	7	7.5
	Adenoid basal-cell carcinoma	5	5.4
	Squamous cell carcinoma	5	5.4
	Nonkeratinized squamous cell carcinoma	4	4.3
	Keratinized squamous cell carcinoma	4	4.3
	Oncocytic carcinoma	2	2.2
	Lymphoepithelial carcinoma	2	2.2
	Micropapillary carcinoma	2	2.2
	Myoepithelial carcinoma	5	5.4
	Pseudo sarcomatoid carcinoma	1	1.1
	Polymorphic low-grade carcinoma	1	1.1
	Undifferentiated carcinoma	1	1.1
	Cystadenocarcinoma	1	1.1
	Malignant melanoma	1	1.1

Benign	Cystadenolymphoma (Warthin's tumour)	46	43
	Pleomorphic adenoma	33	30.8
	Basal-cell adenoma	15	14.0
	Oncocytoma	7	6.5
	Myoepithelioma	3	2.9
	Cystadenoma	2	1.9
	Adenolyphoma	1	0.9

NOS, not otherwise specified.

**Table 3:** Expression and detection of HPV, EBV, and HHV-8 in salivary gland tumours by immunohistochemistry, CISH, and chip technology.

	*n*	Positive	Distribution of malignant cases	Distribution of benign cases	Negative	Not evaluable
HHV-8	190	1 (0.5%)	1	0	187 (98.4%)	2 (1.1%)
EBV-LMP-1	190	4 (2.1%)	3	1	183 (96.3%)	3 (1.6%)
EBER-CISH	190	1 (0.5%)	1	0	187 (98.4%)	2 (1.1%)
p16	190	17 (9%)	14	3	159 (83.5%)	14 (7.5%)
HPV (chip)	25^*∗*^	19 (76%)	12	7	1 (4%)	5 (20%)

^*∗*^Cases with strong nuclear p16 positivity and Warthin's tumours; HHV-8, human herpes virus 8; EBV-LMP-1, Epstein-Barr virus latent membrane protein-1; EBER-CISH, Epstein-Barr virus encoded RNA-chromogenic in situ hybridization; p16, cyclin-dependent kinase inhibitor 2A; HPV, human papillomavirus.

**Table 4:** Distribution of HPV type 16 positive cases in salivary gland tumours (*n* = 19).

	Positive
Adenoid cystic carcinoma	4
Adenocarcinoma NOS	3
Invasive ductal carcinoma	1
Acinus cell carcinoma	2
Adenoid basal-cell carcinoma	2
Warthin's tumour	6
Pleomorphic adenocarcinoma	1
